# Decreased Erythrocyte NA^**+**^,K^**+**^-ATPase Activity and Increased Plasma TBARS in Prehypertensive Patients

**DOI:** 10.1100/2012/348246

**Published:** 2012-08-01

**Authors:** Carlos Ricardo Maneck Malfatti, Leandro Tibiriçá Burgos, Alexandre Rieger, Cássio Luiz Rüdger, Janaína Angela Túrmina, Ricardo Aparecido Pereira, João Lang Pavlak, Luiz Augusto Silva, Raul Osiecki

**Affiliations:** ^1^Department of Physical Education, Midwest State University, Campus Irati, 84500-000 PR, Irati, Brazil; ^2^Departamento de Educação Física e Saúde, Universidade de Santa Cruz do Sul, 96815-900 Santa Cruz do Sul, RS, Brazil; ^3^Departamento de Biologia e Farmácia Laboratório de Biotecnologia e Genética, Universidade de Santa Cruz do Sul, 96815-900 Santa Cruz do Sul, RS, Brazil; ^4^Pharmaceutical Science Master Degree Program, Midwest State University, Campus CEDETEG, 85040-080 Guarapuava, PR, Brazil; ^5^Faculdade Campo Real, Biomedicina, 85015-240 Guarapuava, PR, Brazil; ^6^Departamento de Educação Física, Universidade Federal do Paraná, 80215370 Curitiba, PR, Brazil

## Abstract

The essential hypertension has been associated with membrane cell damage. The aim of the present study is investigate the relationship between erythrocyte Na^+^,K^+^-ATPase and lipoperoxidation in prehypertensive patients compared to normotensive status. The present study involved the prehypertensive patients (systolic: 136 ± 7 mmHg; diastolic: 86.8 ± 6.3 mmHg; *n* = 8) and healthy men with normal blood pressure (systolic: 110 ± 6.4 mmHg; diastolic: 76.1 ± 4.2 mmHg; *n* = 8) who were matched for age (35 ± 4 years old). The venous blood samples of antecubital vein (5 mL) were collected into a tube containing sodium heparin as anticoagulant (1000 UI), and erythrocyte ghosts were prepared for quantifying Na^+^,K^+^-ATPase activity. The extent of the thiobarbituric acid reactive substances (TBARS) was determined in plasma. The statistical analysis was carried out by Student's *t*-test and Pearson's correlation coefficient. A *P* < 0.05 was considered significant. The Na^+^,K^+^-ATPase activity was lower in prehypertensive patients compared with normotensive subjects (4.9 versus 8.0 nmol Pi/mg protein/min; *P* < 0.05). The Na^+^,K^+^-ATPase activity correlated negatively with TBARS content (*r* = −0.6; *P* < 0.05) and diastolic blood pressure (*r* = −0.84; *P* < 0.05). The present study suggests that Na^+^,K^+^-ATPase activity reduction and elevation of the TBARS content may underlie the pathophysiological aspects linked to the prehypertensive status.

## 1. Introduction

The pathogenesis of essential hypertension is poorly understood, although accumulating evidence suggests that genetic and environmental factors are of important etiological relevance [1]. One of the factors involved in the development of essential hypertension is the alteration of cellular sodium metabolism.

In humans, a chronic high-salt diet causes the levels of cardiotonic steroids (CTSs) to rise in the plasma [2], like endogenous ouabain-induced increase of blood pressure in salt-dependent hypertensive rats and in certain patients with essential hypertension [3]. Thus, these CTSs may be involved in the etiology of salt-sensitive hypertension and preeclampsia-induced Na^+^,K^+^-ATPase inhibition in salt-sensitive hypertension [4]. Generally, it is believed that CTSs inhibit the plasma membrane Na^+^,K^+^-ATPase, the sodium pump, leading to an increase in cytosolic Na^+^ concentration. Cell Na^+^ accumulation raises the cytosolic Ca^2+^ concentration through the involvement of the Na^+^/Ca^2+^ exchanger (NCX) and thereby increases contraction in vascular smooth muscle or heart muscle. This sequence of events may lead to hypertension, but the hypothesis has not been critically tested, because little is understood of the function of NCX in these processes [2]. 

It has been suggested that biochemical and biophysical abnormalities of cell membranes [5] may actively participate in the pathogenesis of hypertension [6], and that such abnormalities seem to be involved not only in vascular smooth muscle cells, but also in circulating blood cells [7]. In fact, it has been reported that viscosity and rigidity of erythrocyte membranes are increased in spontaneously hypertensive rats (SHR) and in patients with essential hypertension [8], and that erythrocyte membrane fluidity depends on Na^+^,K^+^-ATPase activity [6]. Interestingly, erythrocyte Na^+^,K^+^-ATPase activity is diminished in hypertensive patients, and enzyme activity is restored to normal by a calcium channel blocker [7]. These findings reinforce the view that alterations in erythrocyte Na^+^,K^+^-ATPase activity are linked to hypertension.

In fact, ion transport alterations found in essential hypertension seem to be closely associated with the concomitant changes in lipid metabolism [8]. Multiple abnormalities in ion transport of red blood cell have been observed in hypertensive animals models [9, 10]. Nevertheless, little attention was paid to the relationship between erythrocyte Na^+^,K^+^-ATPase and lipoperoxidation in prehypertensive patients compared to normotensive status. 

## 2. Methods

### 2.1. Subjects

The present study involved prehypertensive patients and healthy men (*n* = 8) with normal blood pressure (controls) who were matched for age (Table 1).

The adopted criteria for the classification of prehypertensive subjectswas for those with blood pressure ranging from 120 to 139 mmHg systolic and/or 80 to 89 mmHg diastolic blood pressure in accordance with the Seventh Report of the Joint National Committee on Prevention, Detection, Evaluation, and Treatment of High Blood Pressure [11].

Three patients carried out regular antihypertensive medicaments (thiazide diuretics: chlorothiazide 125 mg/d, chlorthalidone 12.5 mg/d, and polythiazide 2 mg/d). Informed consent was obtained for the study in accordance with Resolution 196/96 of the National Council of Health in Brazil, which was approved by local Ethics Committee.

### 2.2. Procedures

All reagents were purchased from Sigma (St. Louis, MO, USA) and all solutions were prepared with type I ultrapure water. After blood pressure measurement (BP; mercury column), the venous blood samples of antecubital vein (5 mL) were drawn in random order to hospital at 9.00 am, and were collected into a tube containing sodium heparin as anticoagulant (1000 UI). The erythrocyte membranes (erythrocyte ghosts) were prepared as described by Niggli et al. [12]. First, the cells were centrifuged at 3000 g for 10 min at 4°C and plasma and buffy coat removed. The erythrocyte were then washed thrice with 0.1 M Tris-HCl buffer, pH 7.4 and lysed with hypotonic (15 mM) Tris-HCl buffer (pH 7.4) for 1 h at 4°C. The erythrocyte ghosts were centrifuged at 15000 g for 30 min at 4°C. The cell membrane pellet was washed repeatedly till turning colourless, using the same buffer, and then suspended in 0.1 M Tris-HCl buffer and homogenized. Aliquots were used for assay of protein. The protein content was measured by method of Bradford [13]. The total Na^+^,K^+^-ATPase activity was assayed at 37°C in an incubation mixture containing 30 mmol/L Tris-HCl, pH 7.4, 0.1 mmol/L EDTA, 50 mmol/L NaCl, 5 mmol/L KCl, 6 mmol/L MgCl_2_, and 1 mmol/L ATP in the presence or absence of ouabain (0.5 mM), as described by Reinila et al. [14]. Briefly, after preincubating the isolated membranes (50 *μ*g) for 10 min at 37°C, the reaction was started by the addition of ATP and stopped with 50 *μ*L of TCA (30%), after 20 min. The amount of inorganic phosphate released was determined by the method of Lanzetta et al. [15] and Na^+^,K^+^-ATPase activity was calculated as the difference between the presence or absence of ouabain-sensitive Na^+^,K^+^-ATPase activity. All reagents were purchased from Sigma (St. Louis, MO).

The extent of lipid peroxidation (TBARS) was determined according to the adapted method of Jentzsch et al. [16]. TBA (25 *μ*L) reagent (0.11 mol/L: 800 mg TBA dissolved in 50 mL 0.1 mol/L NaOH) were added and vortexed again. The reaction mixture was then incubated at 90°C for 45 min in a water bath. The tubes were then put on ice to stop the reaction and followed by centrifugation at 3000 rpm for 15 min. The absorbance of the supernatant was read at 540 nm at room temperature against blank.

### 2.3. Statistical Analyses

All data are expressed as means ± SD. The statistical analyses of hemodynamical (blood pressure) and biochemical (Na^+^,K^+^-ATPase and lipoperoxidation) data were carried out by two-tailed unpaired Student's *t*-test. The Pearson's correlation coefficient was determined for systolic, diastolic, mean blood pressure, lipoperoxidation, and Na^+^,K^+^-ATPase activity. A *P* < 0.05 was considered significant.

## 3. Results

**Figure 1:**
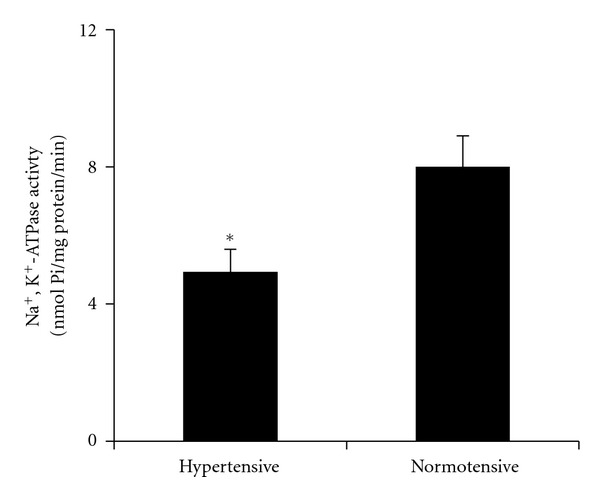
Na^+^,K^+^-ATPase activity (nmol Pi/mg protein/min) in membrane of erythrocyte of hypertensive (*n* = 8) *versus* normotensive (*n* = 8) subjects. *P* < 0.05 by two-tailed unpaired Student's *t*-test.

The Na^+^,K^+^-ATPase activity was lower in patients with prehypertension compared with normotensive subjects [T_14_: 4,  6; *P* = 0.049; (see Figure 1)]. However, the TBARS production was not different between hypertensive and normotensive subjects.

**Figure 2:**
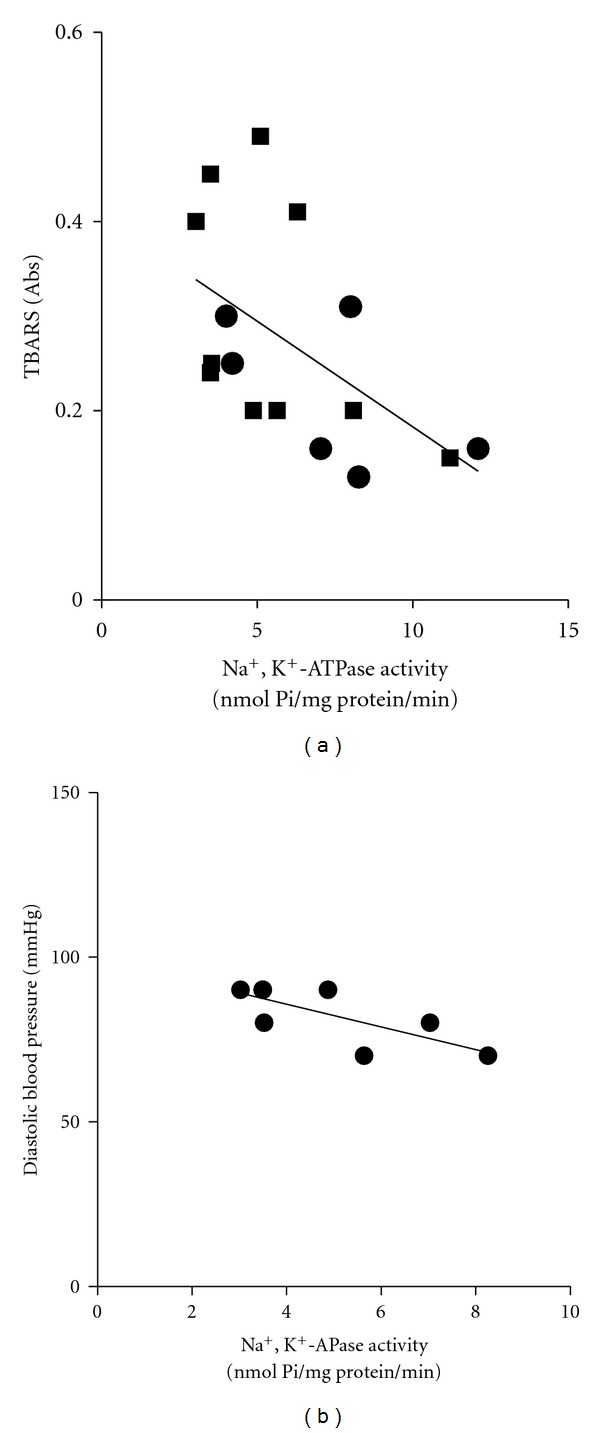
Na^+^,K^+^-ATPase activity (nmol Pi/mg protein/min) negatively correlates with TBARS content only when analyzed with all prehypertensive (■) and normotensive (*⚫*) subjects (*r* = −0.6; *n* = 16) (a) and diastolic blood pressure only in hypertensive subjects (b). *P* < 0.05 by the Pearson's correlation coefficient (*r* = −0.84; *n* = 8).

Figure 2(a) revealed a negative correlation between TBARS and Na^+^,K^+^-ATPase activity only when analyzed with all subjects (normotensive and hypertensive) of study (*r* = −0.6; *P* < 0.05).

Figure 2(b) showed a strong negative correlation between diastolic blood pressure and Na^+^,K^+^-ATPase activity only for hypertensive subjects (*r* = −0.84; *P* < 0.05). The correlation test between systolic and mean blood pressure with Na^+^,K^+^-ATPase activity was not significant.

## 4. Discussion

Interestingly, previous studies have proposed that increased viscosity and rigidity of erythrocyte membranes may contribute to increased peripheral resistance in hypertension [7]. Erythrocyte membrane fluidity depends on Na^+^,K^+^-ATPase activity, which is reduced in hypertensive patients [16] and in spontaneously hypertensive rats [6]. The mechanisms underlying such reduction are still obscure, but it has been reported that plasma membrane Na^+^,K^+^-ATPase may be inhibited by ouabain-like endogenous inhibitors [5], which are decreased by physical exercise [17] or antihypertensive agents, which can enhance Na^+^,K^+^-ATPase activity [10].

Indeed, it has been showed that chronic administration of ouabain caused hypertension in rats, which can be suppressed by SEA0400, an ouabain antagonist [18]. Moreover, ~50% of patients with essential hypertension have substantially elevated levels of endogenous ouabain [2]. Elevated CTSs levels in plasma, like ouabain, can increase the blood pressure by Na^+^,K^+^-ATPase inhibition, [Na^+^] accumulation facilitates Ca^2+^ entry through the vascular contract peripheral blood vessels via vascular Na^+^/Ca^2+^ exchanger (NCX) and thereby result in hypertension [2].

Notably, in vascular smooth muscle (VSM) cells, the NCX1 is colocalized with Na^+^,K^+^-ATPase *α*_2_ and *α*_3_ isoforms, which have high affinity for ouabain [19]. The Na^+^,K^+^-ATPase of human erythrocytes is composed of *α*_1_, *α*_3_, *β*_1_, and *β*_3_ isoforms [20]. It is very possible that the reduced activity of Na^+^,K^+^-ATPase in erythrocyte membranes and its inverse relationship with TBARS content are also occurring in cardiac and VSM cells of patients with prehypertension.

Moreover, alterations of the antioxidant status and increased lipoperoxidation have been also proposed as a cause of Na^+^,K^+^-ATPase reduction in erythrocyte membranes [16]. Regarding this point, it is worth noting that the subjects that showed higher values of lipoperoxidation also had lower values of Na^+^,K^+^-ATPase activity, independent of hypertension diagnostic. It can suggest but cannot prove that an initial damage in cell membranes can lead to future complications, like erythrocyte Na^+^,K^+^-ATPase activity reduction and hypertension development. However, it cannot determine by itself if the diminution of the Na^+^,K^+^-ATPase activity found in the present study is cause or consequence of the hypertension.

In addition, in the present study we observed that the only diastolic blood pressure in the hypertensive subjects (Figure 2) negatively correlated with Na^+^,K^+^-ATPase activity. This finding is particularly remarkable, since the more was Na^+^,K^+^-ATPase reduced, the more subjects presented elevated diastolic blood pressure, evidenced by the significant negative correlation between these variables, which suggests a possible role for Na^+^,K^+^-ATPase reduction in blood pressure increase. Recently, it was published that the aerobic exercise improves erythrocyte Na^+^,K^+^-ATPase activity correlated negatively with blood pressure in spontaneously hypertensive rats [9]. In the present study, the negative correlation between Na^+^,K^+^-ATPase activity and lipoperoxidation suggests that increasing free radicals production may play a role in the currently reported reduction of erythrocyte Na^+^,K^+^-ATPase activity and consequent increase in the blood pressure. 

## 5. Conclusion

In the present study we report that diastolic blood pressure and lipoperoxidation negatively correlated with erythrocyte Na^+^,K^+^-ATPase activity. We suggest that Na^+^,K^+^-ATPase activity reduction and an elevation of lipoperoxidation may underlie one of the pathophysiological aspects linked to the prehypertensive status, but more randomized studies are necessary to clarify this point.

## Figures and Tables

**Table 1:** Clinical characteristics of subjects.

Variable	Normotensive	Hypertensive
	(*n* = 8)	(*n* = 8)
Systolic blood pressure (mmHg)	110.2 ± 6.4	136.8 ± 7^∗^
Diastolic blood pressure (mmHg)	76.1 ± 4.2	86.8 ± 6.3^∗^
Mean blood pressure (mmHg)	91.25 ± 5.01	100.83 ± 4.9^∗^
Age (years)	36 ± 3	34 ± 5
Pharmacological therapy	0	3

*Significant difference at *P* < 0.05. Values are means ± SD.
